# COMPARTMENTAL FEMUR CORTICAL THICKNESS IN OLDER ADULTS DIFFERS BY DEMOGRAPHICS AND PHYSICAL FUNCTION

**DOI:** 10.1093/geroni/igac059.2833

**Published:** 2022-12-20

**Authors:** Justin Lam, Delanie Lynch, Diana Madrid Fuentes, Karan Devane, Marjorie Howard, Kristen Beavers, Ashley Weaver

**Affiliations:** Carnegie Mellon University, Pittsburgh, Pennsylvania, United States; Wake Forest School of Medicine, Winston Salem, North Carolina, United States; Wake Forest School of Medicine, Winston Salem, North Carolina, United States; Wake Forest University Health Sciences, Winston-Salem, North Carolina, United States; Wake Forest School of Medicine, Winston Salem, North Carolina, United States; Wake Forest University Health Sciences, Winston-Salem, North Carolina, United States; Wake Forest School of Medicine, Winston Salem, North Carolina, United States

## Abstract

Previous studies have examined the relationship between cortical bone properties and fracture risk in older populations, but they have yet to examine how these properties change within the femur. Most hip fractures are classified by their location: femoral neck, intertrochanteric, or subtrochanteric fractures. Since cortical thickness can vary throughout the femur, a quantitative method of examining thickness by region was developed using a cortical mapping approach applied to computed tomography (CT) scans. Subject-specific finite element (FE) models of proximal femurs were created from baseline CT scans of 107 older adults (ages 60 – 85; 70% White; 72% Female) with obesity as classified by BMI (33.8 ± 4 kg/m2). An existing FE model was morphed to the segmented geometry of each subject’s proximal femur. A nearest neighbor search assigned cortical thickness values to the nearest finite element model node. Cortical thickness was grouped into four femoral compartments: femoral head, femoral neck, intertrochanteric and subtrochanteric regions. Pairwise paired t-tests indicated that cortical thickness differed between femoral compartments (p < 0.05). Multivariate regression models showed greater cortical thicknesses in femoral head, neck, and intertrochanteric regions in African Americans compared to Whites (p < 0.05). Additionally, these models suggest an association between cortical thickness and physical function assessments, such as the timed up-and-go test and leg muscle strength test. Since cortical thickness is not constant throughout the femur, future studies can use the framework developed here to assess each compartment individually and investigate the relationships between cortical thickness, demographics, and functional assessments.

